# Nitric oxide inhibits ten-eleven translocation DNA demethylases to regulate 5mC and 5hmC across the genome

**DOI:** 10.21203/rs.3.rs-4131804/v1

**Published:** 2024-04-03

**Authors:** Douglas Thomas, Marianne Palczewski, Hannah Kuschman, Brian Hoffman, Hao Yang, Sharon Glynn, David Wilson, Eric Kool, William Montfort, Jenny Chang, Aydolun Petenkaya, Constantinos Chronis, Thomas Cundari, Sushma Sappa, Kabirul Islam, Daniel McVicar, Yu Fan, Qingrong Chen, Daoud Meerzaman, Michael Sierk

**Affiliations:** University of Illinois Chicago; University of Illinois Chicago, College of Pharmacy, Department of Pharmaceutical Sciences; University of Illinois Chicago, College of Pharmacy, Department of Pharmaceutical Sciences; Northwestern University; Weinberg College of Arts and Sciences, Northwestern University, Department of Chemistry; University of Galway, College of Medicine, Nursing and Health Sciences, School of Medicine, D. of Pathology; Stanford University; Stanford University, Department of Chemistry, School of Humanities and Sciences; University of Arizona, Department of Chemistry and Biochemistry; Houston Methodist, Department of Medicine and Oncology, Weill Cornell Medical College; University of Illinois Chicago, College of Medicine, Biochemistry and Molecular Genetics; University of Illinois Chicago, College of Medicine, Biochemistry and Molecular Genetics; University of North Texas, Department of Chemistry; University of Pittsburgh, Department of Chemistry; University of Pittsburgh; National Institutes of Health, National Cancer Institute, Center for Cancer Research; National Cancer Institute, Center for Biomedical Informatics and Information Technology; National Cancer Institute, Center for Biomedical Informatics and Information Technology; National Cancer Institute, Center for Biomedical Informatics and Information Technology; National Cancer Institute, Center for Biomedical Informatics and Information Technology

## Abstract

DNA methylation at cytosine bases of eukaryotic DNA (5-methylcytosine, 5mC) is a heritable epigenetic mark that can regulate gene expression in health and disease. Enzymes that metabolize 5mC have been well-characterized, yet the discovery of endogenously produced signaling molecules that regulate DNA methyl-modifying machinery have not been described. Herein, we report that the free radical signaling molecule nitric oxide (NO) can directly inhibit the Fe(II)/2-OG-dependent DNA demethylases ten-eleven translocation (TET) and human AlkB homolog 2 (ALKBH2). Physiologic NO concentrations reversibly inhibited TET and ALKBH2 demethylase activity by binding to the mononuclear non-heme iron atom which formed a dinitrosyliron complex (DNIC) preventing cosubstrates (2-OG and O_2_) from binding. In cancer cells treated with exogenous NO, or cells endogenously synthesizing NO, there was a global increase in 5mC and 5-hydroxymethylcytosine (5hmC) in DNA, the substrates for TET, that could not be attributed to increased DNA methyltransferase activity. 5mC was also elevated in NO-producing cell-line-derived mouse xenograft and patient-derived xenograft tumors. Genome-wide DNA methylome analysis of cells chronically treated with NO (10 days) demonstrated enrichment of 5mC and 5hmC at gene-regulatory loci which correlated to changes in the expression of NO-regulated tumor-associated genes. Regulation of DNA methylation is distinctly different from canonical NO signaling and represents a novel epigenetic role for NO.

## Introduction

DNA methylation at the 5-carbon position of cytosine bases (5mC) is propagated through cell division and is considered a key epigenetic mechanism for cellular memory of transcriptional states. 5mC at promoter CpG islands has been traditionally associated with repression of gene expression, X-chromosome inactivation, and genomic imprinting. Recent research has demonstrated a more complex relationship between DNA methylation and gene expression, that differs depending on the genomic context in which it occurs and is not restricted to transcriptional regulation/silencing at promoter regions. For example, 5mC methylation at enhancer regions can repress enhancer activity whereas gene body methylation can facilitate transcriptional elongation and is associated with actively transcribed genes.

Regardless of the functional consequences of 5mC on gene expression, specific DNA methylation states are established and maintained in cells by the concerted activities of specific methyl-modifying enzymes. This enzymatic machinery includes DNA methyltransferases (DNMTs), which install 5mC on DNA, and Ten-Eleven Translocation (TET) enzymes that facilitate active demethylation. TET enzymes, of which there are 3 isoforms (TET1, TET2, and TET3), sequentially oxidize 5mC to 5-hydroxymethylcytosine (5hmC), 5-formylcytosine (5fC) and 5-carboxylcytosine (5caC) followed by thymine DNA glycosylase (TDG)-dependent base excision repair which results in an unmethylated cytosine. Emerging studies indicate that these more oxidized forms of 5mC (i.e., 5hmC, 5fC, and 5caC) can similarly participate in transcriptional activation or repression when located within gene bodies or other non-promoter genomic regions.

Despite the important gene-regulatory functions of DNA methylation, pathological alkylation damage from exposure to environmental or therapeutic alkylating agents or endogenous metabolic processes also causes DNA methylation. This type of alkylation DNA damage methylation occurs on any of the 4 DNA bases, but it is most prevalent on adenine (as 1-methyladenine (1mA)) and on cytosine (as 3-methylcytosine (3mC)). To safeguard genomic integrity, repair pathways have evolved to recognize these cytotoxic alkylation-induced DNA lesions. ALKBH2 is a DNA repair enzyme in the AlkB family of enzymes that recognizes and demethylates 1mA and 3mC back to adenine and cytosine, respectively. CpG 5mC-DNA methylation and alkylation-mediated DNA methylation (1mA and 3mC) are both associated with numerous disease states including cancers. In cancer, hyper- or hypo-5mC DNA methylation can deactivate tumor suppressor genes or activate oncogenes, while 1mA and 3mC are mutagenic.

Despite the importance of methylated DNA bases and their oxidized derivatives in controlling normal gene expression or facilitating oncogenic transcriptional states, there is a lack of mechanistic knowledge regarding endogenous molecular regulators of DNA methyl-modifying machinery. Nitric oxide (NO) is an endogenously produced free radical signaling molecule that regulates protein function by either directly binding metal centers (the mechanism that activates soluble Guanylate Cyclase (sGC)) or by forming S-nitrosothiols (RSNO) on cysteine residues. These signaling mechanisms underlie many NO-mediated physiological functions (i.e., immune defense, neurotransmission, and vasodilation). Nevertheless, the current understanding of NO signaling does not sufficiently account for its diverse effects, especially in pathological conditions where NO synthesis and signaling are perturbed. This is particularly relevant in cancer, in which upregulated NO production, due to the induction of the inducible form of nitric oxide synthase (NOS2), is associated with altered tumor gene expression, poor patient outcomes, increased mortality, and resistance to chemotherapy across cancer types^1^, including triple-negative breast (TNBC)^2–12^, lung^13–15^, prostate^16,17^, brain^18^, colon^19,20^, melanoma^21–23^, and liver^24,25^.

Although dysregulated NO production and perturbations in DNA methylation patterns are both associated with many of the same pathologies, a causal mechanism linking NO synthesis to altered DNA methylation patterns has not been described. Our prior work demonstrated a novel role for NO as an endogenous epigenetic regulator of gene expression by controlling histone post-translational modifications^26,27^ and mRNA methylation (m^6^A) patterns^28^. Mechanistically, we demonstrated that physiologic NO concentrations could inhibit the catalytic activities of both histone lysine demethylases (KDM) and the RNA demethylase (fat mass and obesity associated protein (FTO)^29–31^). As KDM, FTO, TET, and ALKBH2 all belong to the same family of Fe(II)/2-oxoglutarate(OG)-dependent oxygenases, we hypothesized that NO would similarly inhibit TET and ALKBH2 DNA demethylases. The catalytic activities of all Fe(II)/2-OG-dependent demethylases require the sequential binding of 2-OG and then molecular oxygen (O_2_) to the mononuclear iron atom in the active site. Herein, we demonstrate that TET and ALKBH2 DNA demethylase activity is inhibited by the binding of two molecules of NO to the catalytic iron during the resting state of enzymes forming a dinitrosyliron complex (DNIC).

In this study we focused on models of TNBC, as patients with this molecular subtype that harbor NOS2-expressing tumors have a significantly higher mortality rate than patients with tumors that do not express NOS2. We demonstrate that cells exposed to endogenous or exogenous physiologic NO concentrations exhibit inhibition of TET demethylation that causes gene-specific enrichment of 5mC/5hmC at known tumor-permissive gene-regulatory loci. Although the ability to correctly assign the transcriptional regulation of a particular gene to the local (or distant) presence of DNA methylation has remained surprisingly limited^32^, our data suggest that deleterious phenotypic effects of NO are partially determined by transcriptional reprogramming mediated by TET inhibition and the resultant changes in the DNA methyl landscape.

## Results

### Determination of NO as an inhibitor of TET and ALKBH2 demethylase activity *in vitro*.

**Figure 1:**
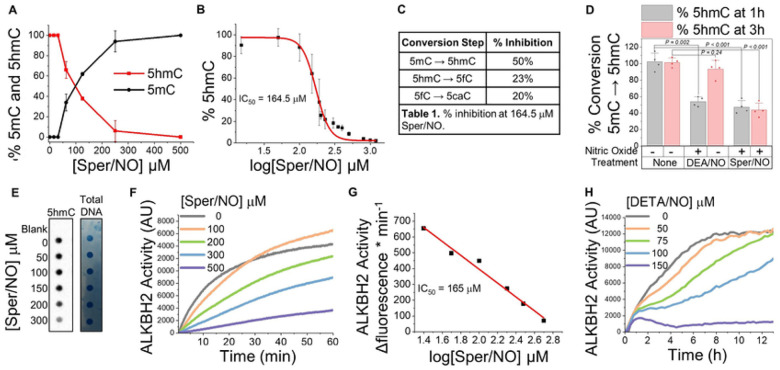
NO inhibits Fe(II)/2-OG-dependent DNA demethylase activity. **(A–E)** TET2 demethylase activity **(A)** Conversion of 5mC (black) to 5hmC (red) generated when human wt-TET2 was incubated with NO (Sper/NO; 0–500 mM; 3h). Demethylase activity assay was initiated with addition of 5mC-double-stranded DNA substrate. **(B)** TET2 activity (5hmC formation) measured over a range of Sper/NO concentrations (0 – 1.5 mM), IC_50_=164.5 mM Sper/NO (~0.5–2 mM NO). **(C)** % inhibition: TET2 was incubated with 164.5 mM Sper/NO and with different starting DNA substrates for TET2 (5mC, 5hmC, or 5fC) for 3 h. **(D)** TET2 demethylase activity (product formation (5hmC)) was measured at 2 time points, 1 hour (gray bars) and 3 hours (red bars). Activity was measured on TET2 alone or after incubation with NO for different lengths of time using two NO-donors (rapid NO release: DEA/NO, and (slower NO release: Sper/NO). ELISA, n=4. **(E)** TET2 activity in the presence of freshly isolate genomic DNA (1 mg) and NO (Sper/NO 0–300 mM), 5hmC measured by dot-blot hybridization using anti-5hmC antibody and total DNA measured by methylene blue. **(F–H)** ALKBH2 activity. **(F)** Recombinant ALKBH2 was incubated with Sper/NO (0–500 mM) and the fluorescent probe. Demethylation of the fluorescent probe (ALKBH2 activity) was continuously monitored by fluorescence spectroscopy at 480 nm for 1 h. **(G)** IC_50_ determination from data in “F”. **(H)** Cellular extracts containing ALKBH2 were incubated with the fluorescent probe and the slow NO-releasing donor DETA/NO (t_1/2_=22h, 0–150 mM), demethylation was measured in real time for 12 h. Data are Mean ± S.E.M, n≥3.

In humans, active DNA demethylation is catalyzed by 2 members of the Fe(II)/2-OG-dependent family of oxygenases; TET (there are 3 isoforms: TET1, TET2 & TET3) and ALKBH2. To test whether NO could inhibit TET catalytic activity we incubated the purified catalytic domain of human TET2 enzyme with all cofactors (2-OG, Fe(II), and ascorbate), substrate (synthetic 5mC-DNA oligo), and with NO (the NO-donor Sper/NO, 50 – 500 mM). After 1 hour incubation we measured the relative amounts of the substrate for TET2 (5mC) and its initial oxidation product (5hmC) at each NO (Sper/NO) concentration using a MALDI-TOF-MS-based assay^33^ (Fig. 1A). Using a broader range of physiologic/pathologic NO concentrations, we determined that NO inhibited TET2 demethylase activity with half-maximal inhibitory concentration (IC_50_) of 164.5 mM for Sper/NO (~1.5 mM NO) (Fig. 1B). Next, using the IC_50_ concentration for Sper/NO, we tested whether NO could also inhibit the stepwise TET2-catalyzed oxidation of 5hmC to 5fC and, in a separate reaction, the conversion of 5fC to 5caC. When synthetic DNA oligos containing 5hmC were used as TET2 substrates, the enzymatic conversion of 5hmC to 5fC was inhibited by 23%. Using 5fC as the substrate, conversion to 5caC was inhibited by 20% (Fig. 1C).

To determine how the duration of NO exposure affected TET2 activity and if TET2 inhibition by NO was reversible, we compared the demethylase activity of TET2 alone with the activity of TET2 incubated with NO for either a short or a long exposure time. This was accomplished using DEA/NO, a short-acting NO donor (t_½_ ≈ 2 min at 37° C), or Sper/NO, a longer-acting NO donor (t_½_ ≈ 37 min at 37 °C)^34^. By using the same concentrations of these two NO donors, we were able to expose the enzyme to the same amounts of NO (moles) but for different durations of time. TET2 activity was measured at 1 and 3 hours. When TET2 was not treated with NO, demethylation of 5mC to 5hmC was 100% complete within one hour (“None”, Fig. 1D). When TET2 was incubated for a short period of time with NO, its demethylase activity was initially inhibited (~50% product formation at 1 h), but when NO was no longer present, TET2 was able to completely convert 5mC to 5hmC at 3 hours (“DEA/NO”, Fig. 1D). Conversely, when TET2 was exposed to a continuous steady-state NO concentration for the duration of the experiment, the catalytic activity of TET2 was inhibited (~55%) within 1 hour and no further catalysis occurred during the remaining 2 hours of the experiment (“Sper/NO”, Fig. 1D). These results suggest that TET2 inhibition by NO is reversible. Next, instead of using a synthetic 5mC-DNA oligo substrate, we tested NO-mediated TET2 inhibition using its biological substrate: genomic DNA (Fig. 1E). Again, NO inhibited TET2-catalyzed conversion of 5mC to 5hmC in a concentration-dependent manner.

Having established that NO could inhibit TET2, we tested whether NO could similarly inhibit ALKBH2, another Fe(II)/2-OG-dependent DNA demethylase. ALKBH2 activity was measured in real-time using a fluorescence assay^35^. This assay used a fluorogenic 1-methyladenine probe that has a >10-fold increase in fluorescent signal intensity when the alkyl group is demethylated by ALKBH2. We incubated recombinant ALKBH2 and all cofactors (2-OG, Fe(II), ascorbate) with the methylated probe substrate and NO (Sper/NO, 100–500 mM) for 60 minutes (Fig. 1F). Under these conditions, NO inhibited ALKBH2 demethylase activity in a concentration-dependent manner with an IC_50_ for Sper/NO of 165 mM (Fig. 1G). Next, we isolated nuclear and cytosolic extracts containing endogenous ALKBH2 from cultured MDA-MB-231 breast cancer cells to test whether NO could similarly inhibit ALKBH2 derived from biological sources. The combined extracts were exposed to low steady-state concentrations of NO using the NO donor DETA/NO (t_½_ ≈ 22 h, 50–150 mM) and demethylase activity was monitored for 12 h using the fluorescent probe method (Fig. 1H). Over this range of DETA/NO concentrations we expect that the steady-state concentrations of NO correspond to low nM physiological levels (as we have previously measured^36,37^). Under these conditions ALKBH2 demethylase activity was inhibited in a dose-dependent manner. Collectively, these results indicate that NO is a potent inhibitor of Fe(II)/2-OG-dependent DNA demethylases TET2 and ALKBH2. For subsequent studies we solely focused on TET enzymes because of their gene regulatory functions (rather than ALKBH2 which is part of the DNA damage response).

### Nitric oxide forms a dinitrosyl iron complex at the mononuclear non-heme iron atom in TET2.

**Figure 2:**
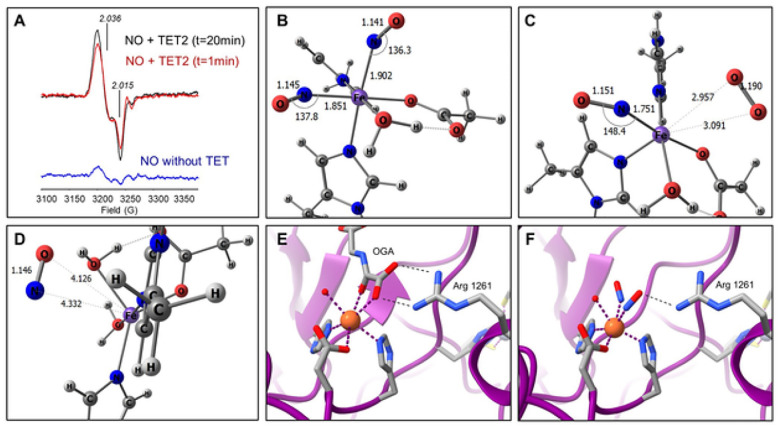
NO forms a DNIC at the mononuclear non-heme iron atom of TET2. **(A)** Representative X-Band (77K) EPR spectra of full length TET2 protein treated with Sper/NO (100 mM) and all substrates and cofactors for 1 min (red line) and 20 min (black line). Control reaction (blue line) is the complete reaction without TET2 after 20 min, n=3. Spectrum is indicative of a non-Heme NO-bound DNIC. **(B)** DFT computations. Core of the wB97xD/def2-svp optimized geometry of triplet di-nitrosyl complex [Fe^II^(OAc)(Im)_2_(NO)_2_(OH_2_)]^+^, OAc = acetate, Im = N-methyl-imidazole. Bond lengths and angles in Å and °, respectively. **(C)** Core of the wB97xD/def2-svp optimized geometry of [Fe(OAc)(Im)_2_(NO)(OH_2_)_2_]^+^ and **(D)** [Fe(OAc)(Im)_2_(NO)(O_2_) (OH_2_)]^+^. **(E)** Crystal structure of TET2 in complex with N-oxalyglycine (OGA), a 2-OG analog (PDB file 4nm6). OGA forms two critical hydrogen bonds with a conserved arginine (Arg 1261 in TET2). **(F)** Two NO molecules replace the two oxygens from OGA that coordinate with Fe(II).

A critical step in the TET-catalyzed DNA demethylation reaction is binding O_2_ to the non-heme iron atom^38^. We hypothesized that because of NO’s structural and bonding similarity to O_2_ that NO would compete with O_2_ and inhibit TET by forming the more stable mononitrosyl complex at the iron site. To test whether NO interacts with the iron center, we conducted electron paramagnetic resonance (EPR) studies of TET2 treated with NO in the presence of all cofactors and substrate. We ran two identical reactions in parallel and stopped them at different time points (<1 and 20 minutes). EPR spectra at both time points showed that the enzyme does not form the expected S = 3/2 mononitrosyl complex (Fe(II)-NO), as shown by the absence of its intense g_┴_ ~ 4.1 signal (**Fig. S1A**), and instead revealed the characteristic S = ½ signal of a non-heme dinitrosyl iron complex (DNIC, [Fe(NO)_2_]^9^), with g_┴_ ~ 2.03, g_||_ ~ 2.01 (Fig. 2A)^37,39,40^. The signature EPR spectrum of DNIC was almost undetectable when the TET2 enzyme was omitted from the complete reaction mixture (**Fig. S1A** & 2A), indicating that the observed DNIC signal is associated with the enzyme, and not with a complex formed in solution. Kinetically, the reaction was ~80% complete by the time all reactants were added, as shown by the small increase in the EPR intensity between samples frozen at 1 min and 20 min.

### Density functional computations support the formation of a DNIC at the catalytic iron atom in TET2.

Because the DNIC was formed instead of the mononitrosyl a series of density functional theory (DFT) computations were performed to investigate the relative affinity for binding of NO versus O_2_ and water in a TET2 resting state. An active site model of TET2 was generated akin to that reported by Lu *et al*.^41^ The charge of the model DNIC complex was adjusted to yield a neutral {Fe(NO)_2_}^9^ electron count, *i.e*., d^6^ Fe(II) + 2 NO p* e^−^ + 1 additional e-. Additional simulations were performed to assess NO binding to models in which 2-OG was ligated to the inner coordination sphere, but these were largely inconclusive apart from indicating that NO is bound more weakly to Fe(II) after ligation of 2-OG. The iron in the enzyme is coordinated by two histidine (His) and one aspartate (Asp) residue. To mimic the pertinent amino acid side chains, Asp was modeled by an acetate (OAc^−^) and His was modeled by N-methylimidizaole (Im). It was assumed that Asp and His model ligands would maintain a fac configuration.

The neutral {Fe(NO)_2_}^9^ dinitrosyl complex Fe(OAc)(Im)_2_(NO)_2_(OH_2_) was chosen for modeling studies; the geometry is shown (Fig. 2B). Fe(OAc)(Im)_2_(NO)_2_(OH_2_) is predicted to be a triplet with *cis* nitrosyl ligands. The lowest energy coordination isomer had one NO trans to OAc and the other trans to Im. The OAc that mimics the Asp side chain forms a strong hydrogen bond with the ligated water. Given the experimental spectroscopic observations, it was investigated whether one of the NO ligands of Fe(OAc) (Im)_2_(NO)_2_(OH_2_) could be displaced by either water or dioxygen; thus, computed DFT ground states of Fe(OAc)(Im)_2_(NO)(OH_2_)_2_ and Fe(OAc)(Im)_2_(NO)(O_2_)(OH_2_) were sought. The lowest energy geometries are shown in Fig. 2C. In both cases, geometry optimizations initiated from six-coordinate, pseudo-octahedral starting guesses yielded minima with a weakly bound exogenous ligand. For Fe(OAc)(Im)_2_(NO)(OH_2_)_2_, the NO ligand is ejected from the inner coordination sphere upon geometry optimization, Fe⋯NO = 4.13 Å. For Fe(OAc)(Im)_2_(NO)(O_2_)(OH_2_), the complex barely maintains an octahedral geometry with the dioxygen very weakly bonded (Fe⋯O_2_ ~ 2.95 Å). The tenuous nature of O_2_ binding in Fe(OAc)(Im)_2_(NO)(O_2_)(OH_2_) is further indicated by the near complete lack of any spin delocalization from the O_2_ to the complex (r_spin_(O_2_) = 1.99 e^−^) and the computed free energy for NO/H_2_O exchange, DG = +23.0 kcal/mol, indicating that NO binds much more tightly than water. The NO/O_2_ exchange free energy is essentially thermoneutral, DG = −0.3 kcal/mol. In conjunction with the weak O_2_ binding indicated by the long Fe⋯O_2_ bond length of the optimized geometry of Fe(OAc)(Im)_2_(NO)(O_2_)(OH_2_), (Fig. 2D), the DFT results suggest that O_2_ does not readily displace NO, perhaps except at high O_2_ partial pressures, and rationalizes the presence of only the di-nitrosyl complex as seen in the EPR spectra (Fig. 2A).

Based on the DFT results, further modeling was done for the DNIC core and the crystal structure of TET2 in complex with N-oxalyglycine (OGA), a 2-oxoglutarate analog^42^ (PDB ID 4NM6, Fig. 2D). In the model, the NO molecules replace OGA and occupy similar positions to the OGA coordinating oxygens. One of these is close to Arg 1261, which may hydrogen bond to the NO and stabilize overall negative charge buildup on the NO ligands in the DNIC (Fig. 2E,F).

### Nitric oxide increases 5mC in the DNA of human cancer cells.

**Figure 3:**
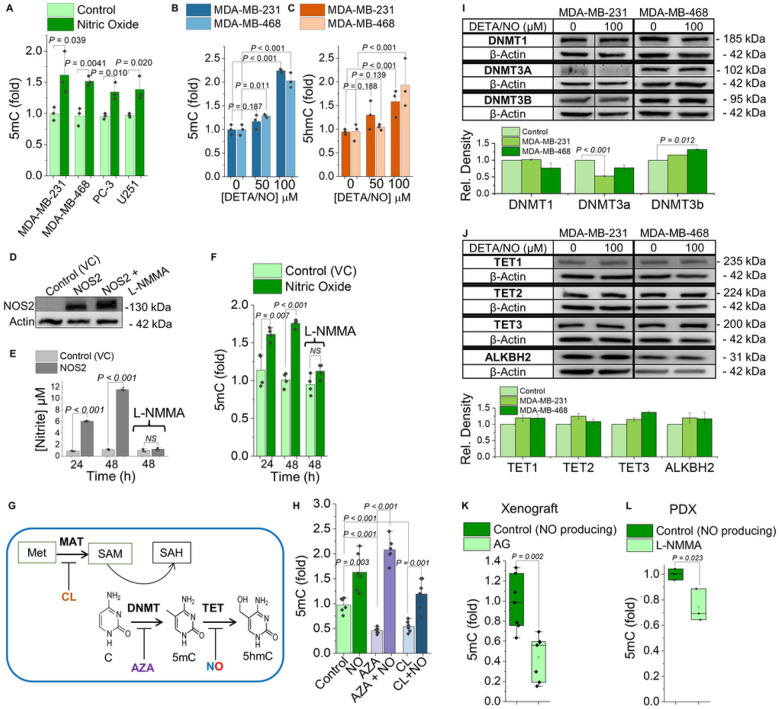
NO increases 5mC on DNA from cancer cells and tumors. **(A)** Relative abundance of 5mC-DNA in MDA-MB-231, −468 (TNBC), PC-3 (prostate), and U251 (glioblastoma) cells that were treated without (light bars) or with (dark bars) NO (DETA/NO 100 mM, 24 h), ELISA. **(B)** Relative 5mC-DNA and **(C)** Relative 5hmC-DNA from MDA-MB-231 and MDA-MB-468 cells that were chronically treated with NO for 10 days (DETA/NO 50 and 100 mM added every 48 h), ELISA. **(D)** Immunoblot for NOS2 protein in MDA-MB-231 empty vector control (VC), MDA-MB-231 NOS2-expressing, and MDA-MB-231 NOS2 + L-NMMA (NOS inhibitor, 1 mM) cells after 24 h. **(E)** Total NO synthesis as measured by accumulation of nitrite (a stable NO oxidation product) in the media after MDA-MB-231 (VC) and MDA-MB-231 NOS2 +/− L-NMMA cells were cultured for 24 and 48 hours. **(F)** 5mC-DNA (same conditions as panel “E”), ELISA. **(G)** Schematic showing how inhibitors cycloleucine (CL), 5-Azacytidine (AZA), and NO interact with DNMT (DNA methyltransferase), MAT (methionine adenosyltransferase), and TET to affect 5mC-, 5hmC-DNA levels. **(H)**5mC-DNA from MDA-MB-231 cells treated for 24 h +/− NO (100 mM DETA/NO), and +/− 1 mM 5-Azacytidine (AZA), or +/− 1 mM cycloleucine (CL), ELISA. **(I, J)** MDA-MB-231 and MDA-MB-468 cells were cultured for 10 days with NO (DETA/NO, 100 mM). **(I)** The expression levels of DNA methyltransferases (DNMT1, 3A, and 3B) and **(J)** DNA demethylases (TET1,2, and 3, ALKBH2) were measured in both cell lines by immunoblotting. Densitometry for protein expression levels in each cell line relative to paired untreated control cell, n = 3 separate experiments. (thin black vertical line indicates splicing of lanes that were run on the same gel but were noncontiguous). **(K)** 5mC-DNA levels in MDA-MB-231 xenograft tumors from mice with”NO-producing” tumors (control; dark green bars), and from mice with tumors that did not synthesize NO (treated with a NOS2 inhibitoraminoguanidine (AG) for 37 days, light bars), ELISA, n=7/group. **(L)** 5mC-DNA from TNBC PDX tumors from control mice with tumors that did synthesize NO (control, vehicle saline injection; dark green bars) and from tumors that did not synthesize NO(administered the pan-NOS inhibitor NG-monomethyl-L-arginine (L-NMMA)), ELISA, n=3/group. Data are represented as mean ± S.E.M., P-values were determined by unpaired two-tailed Student’s t-tests, NS = not significant, n ≥ 3.

Having established that NO was a direct and potent inhibitor of TET enzymes under isolated conditions, the next step was to investigate whether NO could inhibit endogenous cellular TET enzymes and to determine if this would regulate nuclear DNA methylation. We selected four human cancer cell lines derived from aggressive tumor types that are known to express *NOS2* and synthesize NO *in vivo* (2 triple negative breast (TNBC), 1 prostate, 1 brain). These cells do not synthesize NO in culture, so we treated them with DETA/NO (100 mM; 24 h), which resulted in low nM concentrations of NO, and measured 5mC-DNA (Fig. 3A). In all cell lines NO significantly increased global 5mC in DNA. Among NO-associated cancers, TNBC patients who harbor NOS2-expressing tumors have significantly worse prognoses. For this reason, we conducted all subsequent experiments using models of TNBC.

Under biological conditions, DNA methylation patterns are faithfully maintained over multiple cell generations as a form of epigenetic inheritance. Therefore, we developed a cell model to study DNA methylation responses to NO after multiple cell generations; this more accurately mimics the microenvironment of NOS2-expressing tumors *in vivo* where cells are exposed to chronic NO synthesis. We treated two TNBC cell lines with low physiologic steady-state concentrations of NO for 10 days (~12 cell doublings (NO did not alter the doubling rate)) and examined long-term “heritable” DNA methylation patterns. In the NO-treated cells, there was a significant increase in 5mC in DNA (Fig. 3B), and an increase in 5hmC in DNA, the first oxidation product of TET (Fig. 3C). Cells not treated with NO had no change in 5mC/5hmC, suggesting 5mC increases were not attributable to epigenetic drift. To mimic the endogenous NO production observed in tumors, we transfected MDA-MB-231 cells with a human NOS2 gene (or empty vector control (VC)) (Fig. 3D). Accumulative NO synthesis was measured after 24 and 48 hours in both cell lines and NO was only detected in the NOS2-transfected cells, not in the cells transfected with the empty vector plasmid or in the NOS2-transfected cells treated with a pan-NOS inhibitor (L-NMMA) (Fig. 3E). 5mC in DNA was also measured in both cell lines at 24 and 48 hours and 5mC was elevated only in the NO-producing cells but not the control cells or cells treated with L-NMMA (Fig. 3F).

5mC is catalytically installed on DNA by DNA methyltransferase enzymes (DNMT) which, along with TET enzymes, maintain steady-state 5mC levels. NO-dependent increases in 5mC could therefore be due to increased DNA methyltransferase activity rather than inhibition of TET DNA demethylase activity. To test this, we treated MDA-MB-231 cells with NO and either a DNA methyltransferase 1 (DNMT1) inhibitor (5-Azacytidine (AZA)), or a competitive inhibitor of methionine adenosyltransferase (MAT) (cycloleucine (CL)). CL depletes the cell of S-adenosylmethionine (SAM), the substrate for DNA methyltransferases (Fig. 3G). In cells treated with either AZA or CL alone, a significant reduction in global 5mC levels was observed as expected^43^, but when NO was present during either CL or AZA treatment, 5mC levels remained elevated (Fig. 3H). Another potential explanation for increases in 5mC would be if NO was changing the expression levels of DNA methyl-modifying enzymes (i.e., increasing DNMT or decreasing TET expression). To examine this possibility, we treated two TNBC cell lines with NO for 10 days and measured the protein expression levels of DNA methyltransferases (DNMT1, DNMT3a, DNMT3b) and DNA demethylases (TET1,2,3, and ALKBH2) (Fig. 3I,J). For all enzymes there was almost no change in protein expression in response to NO after 10 days. Together these data further support the hypothesis that NO-mediated increases in 5mC result from inhibition of TET demethylases and are not a result of changes in the expression levels of DNA methyl-modifying enzymes or increased DNA methyltransferase activity.

### Nitric oxide increases 5mC in DNA from tumors *in vivo*.

To investigate whether NO could increase 5mC *in vivo* we used a mouse xenograft model of NOS2-expressing cell-line derived tumors. Mice bearing NOS2-expressing MDA-MB-231 xenograft tumors were divided into two groups; half were treated with aminoguanidine (AG), a selective inhibitor of NOS2, and the other half were treated with saline (control). After 37 days of treatment, the tumors were removed, the DNA extracted, and 5mC-DNA was quantified (Fig. 3K). 5mC in DNA from the NO-producing tumors was significantly greater than in the tumors where NO synthesis was inhibited. As further confirmation that NO regulates 5mC in vivo we measured 5mCDNA in NOS2-positive patient derived xenograft (PDX) tumors (Fig. 3L). In this experiment the control group received a vehicle saline injection, and the treatment group was administered the pan-NOS inhibitor NG-monomethyl-L-arginine (L-NMMA) daily. After 40 days the tumors were excised and 5mC was measured. Again, in the NO-producing PDX tumors, 5mC was significantly higher than in the tumors where NO synthesis was inhibited (Fig. 3L).

### NOS2 expression and NO production drive aggressive cancer phenotypes.

**Figure 4:**
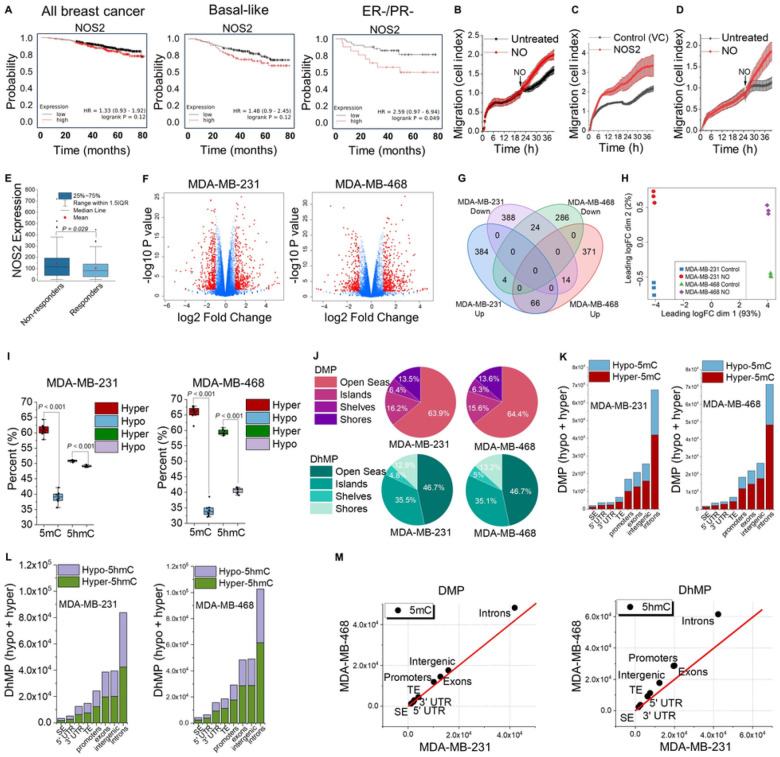
NO causes aggressive cancer phenotypes, regulates tumor-permissive gene expression, and induces loci-specific DNA methylation patterns. **(A)** Patient survival as a function of NOS2 gene expression and hormone receptor status using “Kaplan-Meier Plotter” (public database includes 7,830 unique breast cancer samples). **(B–D)** Cell migration and invasion were measured in real time by the xCELLigence^®^ DP system. **(B)** MDA-MB-231 cells were plated and allowed to adhere for 20 h before the addition of NO (100 mM DETA/NO). **(C)** MDA-MB-231 empty vector control (VC) and MDA-MB-231 NOS2-expressing cells were plated and migration was monitored for 40 h. **(D)** MDA-MB-231 cells were plated on a Matrigel^®^ matrix and incubated for 20 h before the addition of NO (100 mM DETA/NO), n = 6. **(E)** NOS2 gene expression and response to chemotherapy using the ROC Plotter platform which analyzes transcriptomic data sets from TNBC patient tumors (n = 164). **(F–H)** mRNA-Seq was conducted on samples from MDA-MB-231 and MDA-MB-468 cells treated with or without DETA/NO (100 mM; 10 days, n=3 biological replicates). **(F)** Volcano plots of NO-mediated mRNA changes (mRNA-Seq) in MDA-MB-468 and MDA-MB-231 cells cultured with NO compared to untreated control cells. Significantly up- and down-regulated genes are indicated in red, n = 3 biologically independent samples for each cell type (*P*-values, and fold change as indicated). **(G)** Venn diagram demonstrating of the number of significantly NO-regulated genes that overlap between the two cell types. **(H)** Multidimensional scaling (MDS) analysis of gene expression from mRNA-Seq data sets. (**I–M**) Oxidative reduced representation bisulfite sequencing (oxRRBS) was conducted on samples from MDA-MB-231 and MDA-MB-468 cells treated with or without DETA/NO (100 mM; 10 days, n=2 biological replicates). **(I)** Percent of hyper- and hypo-differentially methylated positions (DMP) and hyper- and hypo-differentially hydroxymethylated positions (DhMP) across all annotated sites in both cell types. **(J)** Annotated CpG sites of NO-mediated hyper-DMP and hyper-DhMP in MDA-MB-231 and MDA-MB-468 cells (DMP/DhMP = *P*-*value* < 0.05, mean difference in abs(beta value) of ≥ 0.1 according to RnBeads). **(K)** Gene regulatory locations of NO-mediated hyper- and hypo-differentially methylated CpG positions (DMPs) and **(L)**hyper- and hypo-differentially hydroxymethylated CpG positions (DhMPs) in MDA-MB-231 and MDA-MB-468 cells in response to NO. **(M)** Correlation between the magnitude of DMP and DhMP at functional elements between both cell types. P-values were determined by unpaired two-tailed Student’s t-tests.

Clinically, NOS2 expression in tumors is associated with worse patient outcomes and poor responses to therapy. We used Kaplan-Meier Plotter^44^ to analyze transcriptomic datasets of metastatic breast cancer patients found in GEO, EGA and TCGA. When we examined NOS2 expression in 3 breast cancer patient groups (all subtypes, basal-like, and ER^−^/PR^−^) we found that high NOS2 expression was associated with decreased overall survival (confirming previous reports^11^) (Fig. 4A). To experimentally determine whether NO would result in a more aggressive cell phenotype *in vitro*, we measured cell migration and invasion (two hallmarks of cancer) in real time of TNBC cells exposed to NO. In cells exposed to exogenous NO or in cells endogenously synthesizing NO, the rates of cell migration and invasion were increased compared to untreated control cells (Fig. 4B–D), consistent with what we and others have shown previously^6,45^. Another phenotype associated with tumor aggressiveness is resistance to chemotherapy. Using the ROC plotter platform^46^, we analyzed the expression levels of NOS2 as a predictive biomarker of efficacy of any chemotherapy in TNBC patients (n = 164; response based on relapse-free survival at 5 years). Patient “non-responders” to therapy had significantly higher NOS2 expression than patient “responders” to therapy (Fig. 4E).

### Nitric oxide regulates gene expression in TNBC cells.

With the observation that NO directly inhibits TET demethylase activity leading to global increases in 5mC, we sought to decipher whether this was functionally associated with transcriptional changes in NO-regulated genes that may drive aggressive phenotypes. First, we quantified changes in transcription by performing RNA-sequencing on samples from two TNBC cell lines treated chronically (10 days) with NO. In both cell lines, NO significantly up- and down-regulated several hundred genes compared to untreated control cells (Fig. 4F,G). There were 880 significantly differentially expressed genes in the MDA-MB-231 cells with FDR (False Discovery Rate) <0.05 (454 upregulated log2FC>1, 426 downregulated log2FC <-1) and 765 significantly differentially expressed genes in the MDA-MB-468 cells (451 upregulated log2FC>1, 314 downregulated log2FC <−1). Although there was only a 12 – 14% overlap in common genes transcriptionally regulated by NO between the twOfficell types (90 in total), this may be due to significant differences in their basal transcriptional profiles (control cells not treated with NO). Multidimensional scaling (MDS) analysis illustrated how the expression profiles differed far more between the twOfficell types than between the NO treated and control cells (Fig. 4H). Despite only modest overlap in specific genes transcriptionally regulated by NO in both cell types, Gene Set Enrichment Analysis (GSEA) of the 90 genes differentially expressed in the same direction identified several KEGG pathways relevant to cancer progression (**Table S1**).

### Nitric oxide increases 5mC and 5hmC differentially at specific genomic features.

To determine if NO-mediated changes in 5mC/5hmC were functionally associated with changes in gene expression, we identified the locations of 5mC/5hmC on a genome-wide scale at single-nucleotide resolution by performing oxidative reduced representation bisulfite sequencing (oxRRBS) on samples from two TNBC cell lines that were chronically treated with NO for 10 days. Between the NO-treated cells and the untreated cells we identified differentially methylated positions (DMPs) and differentially hydroxymethylated positions (DhMPs, 5hmC), defined as: p < 0.05 and difference in β-value > 0.1. On a global scale, we found that both 5mC and 5hmC were increasing in both cell types in the NO-treated cells compared to the untreated control cells, consistent with the ELISA data in Figure 3 that NO increases 5mC/5hmC in cells. Although there were net increases in 5mC and 5hmC, these changes were dynamic in that both increases and decreases in 5hmC and 5mC were observed at all annotated regions (Fig. 4I).When we examined annotated CpG sites (islands, shores, shelves, and open seas)^47^ we found that the majority (>60%) of DMPs and (>45%) DhMPs were occurring at open sea positions (Fig. 4J). We then focused on specific functional elements (Super enhancers (SE), 5’ UTR, 3’ UTR, Typical enhancers (TE), promoters, exons, intergenic regions, and introns) to determine if they also exhibited specific patterns of 5mC/5hmC enrichment. In both cell types, and at all genic annotations, we identified hyper- and hypo-DMPs and DhMPs (Fig. 4K, L), with the majority located at introns. The locations of DMPs and DhMPs were similar in both cell types, but the numbers of differentially methylated positions tended to be greater in the MDA-MB-468 cells (Fig. 4M).

### Determination of 5mC- and 5hmC-associated transcriptional changes.

**Figure 5:**
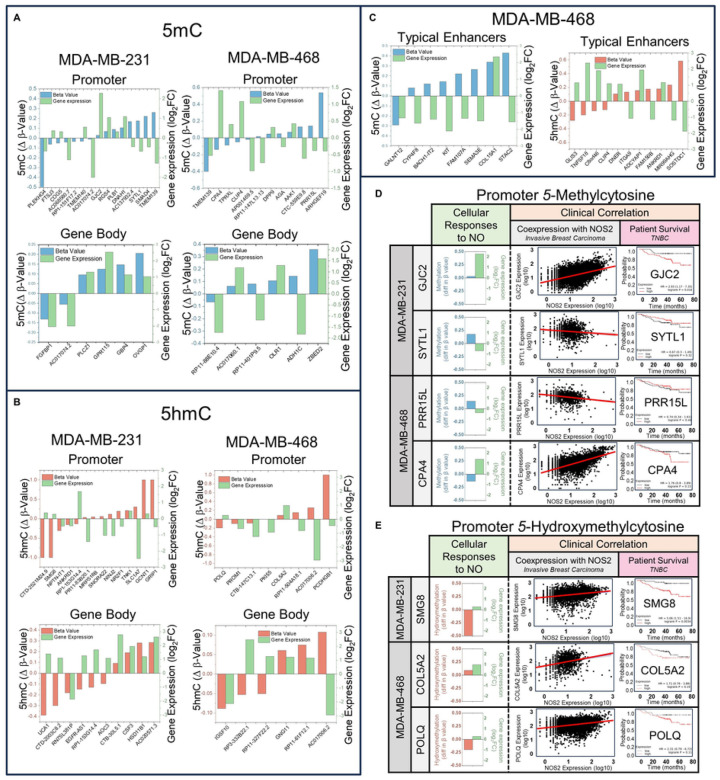
NO-mediated changes in 5mC and 5hmC are associated with transcriptional changes in NO-responsive genes. **(A-C)** b-values (5mC or 5hmC) at gene-regulatory loci (as determined by RnBeads) are ranked from lowest to highest going from left to right and are paired with the expression changes in their associated genes. **(A)** Changes in 5mC (blue) and change in gene expression (green) at promoters and gene bodies for MDA-MB-231 and MDA-MB-468 cells. **(B)**Changes in 5hmC (red) and change in gene expression (green) at promoters and gene bodies for MDA-MB-231 and MDA-MB-468 cells. **(C)** MDA-MB-468 cells changes in 5mC (blue, top) and 5hmC (red, bottom) and change in gene expression (green) at typical enhancers (In MDA-MB-231 cells the enrichment of 5mC/5hmC at typical enhancer regions did not significantly correlate to gene expression changes). **(D)** 5mC-associated genes, and **(E)** 5hmC-associated genes. Left panels “Cellular Responses to NO”, are representative genes shown with their difference in b-value at their promoter region (oxRRBS data) and their expression level (mRNA-seq) in response to NO. Right side “Clinical Correlations”, scatter plot demonstrates correlation between NOS2 expression and specific genes in tumors from patients with aggressive breast cancer. And far right column is correlation between TNBC patient survival and high (red line) or low (black line) expression of the same gene (Kaplan-Meier plot).

Having demonstrated that NO increased 5mC/5hmC in DNA at genomic loci relevant to regulation of gene expression, and that NO produced significant transcriptional changes, we attempted to link changes in DNA methylation to the changes in gene expression. We identified overlaps between significantly expressed genes (RNA-seq) and their b-values (the degree of CpG methylation) at that gene or at gene regulatory loci associated with that gene (i.e. promoters, enhancers, super enhancers). For both control and NO treatment groups there was a clear negative correlation between 5mC b-values at promoters and gene expression in both cell types (**Extended data Fig. 2**). We next identified specific genes that had significant transcriptional changes in response to NO (upregulated or downregulated) and also had significant changes in methylation (increase or decrease in b-value for 5mC or 5hmC) at specific gene-regulatory genomic loci (Fig. 5 A–C, **Extended data Tables 2 & 3**). Although the correlations between methylation status (hyper or hypo, 5mC or 5hmC) of specific gene-regulatory regions and the direction of transcriptional changes were not 100%, certain trends did emerge. For example, increases of 5mC/5hmC at promoters was more associated with downregulated genes whereas gene body enrichment of 5mC/5hmC was more associated with upregulated genes. Increased 5mC/5hmC at typical enhancers correlated to downregulation of associated genes. Although links between many of the differentially expressed genes in Fig. 5 A–C and cancer are unknown or have yet to be established, some of them have an experimental or clinical association with breast cancer progression (Fig. 5D, E). On the left sides of Figures 5D & E (“Cellular Responses to NO”) are select genes that are transcriptionally regulated by NO and show significant changes in their b-values at the promoter regions for these genes (Fig. 5D is **5mC**, 5E is **5hmC**). On the right side of these figures (“Clinical Correlation”) are results from analysis of publicly available data sets of gene expression from tumors of patients with aggressive breast cancers^48^. Genes that were transcriptionally regulated by NO in our cell culture models also had directionally similar gene expression changes in NOS2-expressing patient tumors. Kaplan Meier plots demonstrate the correlation between the NO-regulated gene and patient survival^44^. Although a direct relationship between these NO-regulated genes and cancer progression have yet to be documented in the scientific literature, some reports demonstrate that the genes upregulated by NO (GJC2, CPA4, SMG8, COL5A2, POLQ)^49–53^ and the genes downregulated by NO (SYTL1, PRR15L)^54,55^ are associated with deleterious outcomes when up- or down-regulated in the same direction in cancer patients. These data demonstrate that genes that exhibit changes in their promoter 5mC/5hmC status and are transcriptionally regulated by NO in breast cancer cells in vitro show similar trends in vivo in patient tumors, suggesting a link with NO-regulated genes and the association between poor patient outcomes in breast cancer.

## Discussion

Here we demonstrate that physiologic NO concentrations directly inhibit TET and ALKBH2 via a novel mechanism involving DNIC assembly at the catalytic non-heme iron atom. Moreover, we find that in cancer cells exposed to exogenous NO, or in cells endogenously synthesizing NO, 5mC/5hmC in DNA is increased, and that 5mC and 5hmC are enriched on gene-regulatory loci (i.e., promoter, enhancer regions) of specific NO-regulated genes.

There have been several excellent studies that provided insight and mechanisms through which NO influences DNA methylation patterns^56^, predominantly by modulating the expression levels of DNA methyl-modifying enzymes (i.e., methyltransferases or demethylases). For example, Switzer et al. demonstrated that NO caused a decrease in DNA methylation via S-nitrosation of DNA methyltransferase 1 (DNMT1) which induced DNMT1 degradation^57^. Similarly, another study demonstrated that, in the vascular wall, NO led to an overall decrease in DNA methylation by increasing TET activity and decreasing DNMT activity^58^. Conversely, others have shown that NO enhances the enzymatic activity of DNA methyltransferases (DNMTs) and downregulates TET enzymes to cause an increase in DNA methylation^59^. While our study does not refute these previous findings, we provide multiple lines of biochemical evidence to demonstrate that NO can directly inhibit the catalytic activities of TET enzymes leading to increased 5mC/5hmC on DNA.

There are no structures of NO bound to TET/ALKBH2 and very little data on NO binding to any member of this family of proteins with the exception of two examples of the crystalline form demonstrating formation of the mono-NO at the iron site^60,61^. The non-heme Fe(II) in TET enzymes bind 2-OG in a bidentate manner during their catalytic cycle which leaves one coordination site available for O_2_ binding, or as we initially hypothesize for NO binding. Intriguingly, EPR and modeling studies demonstrated that NO preferentially formed a DNIC and not a mononitrosyl, suggesting NO binds to the enzyme in its resting state prior to 2-OG coordination. This means that NO may compete with 2-OG and not O_2_ to prevent the interaction of TET with all its DNA substrates allowing their differential accumulation. Kinetic experiments, conducted at “room air” oxygen concentrations (~220 mM), revealed that low physiologic concentrations of NO (< nM in the case of ALKBH2) were sufficient to inhibit demethylase activity *in vitro* and *in vivo*. This indicates that TET enzymes have a much higher affinity for NO than O_2_. We also found that NO significantly inhibited TET’s initial oxidation step (5mC Þ 5hmC) more than its subsequent oxidation steps (5hmC Þ 5fC Þ 5caC). Although further mechanistic studies are needed to delineate factors contributing to the differential buildup of TET substrates, these differences may partially be a function of differences in the catalytic rates for each of its oxidation steps, with the first step, conversion of 5mC Þ 5hmC, being the fastest^62^.

Regardless of the mechanism of TET inhibition, *in vitro* (cells culture) and *in vivo* (cell-derived and patient derived xenograft) models demonstrated that NO was associated with global increases in 5mC and 5hmC, which is consistent with other reports demonstrating that TET2,3 knockdown in cancer cells resulted in an increase in 5mC and 5hmC^63^. Moreover, since TET inhibition by NO is reversible this suggests that NO could act as a “molecular switch” to turn on and off demethylase activity as a mechanism of dynamic regulation of DNA methylation. Cellular expression of NOS isoforms, which synthesize NO at different rates, could be the key determinant of whether NO signals epigenetically, through changes in DNA methylation, or predominantly through canonical mechanisms.

To our knowledge, these are the first studies to look at transcriptional changes in cells exposed to chronic (10 day) physiologic low steady-state NO concentrations which more accurately reflects the cellular microenvironment of NOS2-expressing tumors where NO synthesis is constitutively turned on. Although we measured thousands of genes that were transcriptionally changing in response to NO, the overlap between specific genes in two transcriptionally diverse cell types (MDA-MB-231, -468) was relatively small. A major question is whether TET inhibition by NO to increase 5mC/5hmC is a specific mechanism to mediate these transcriptional changes or whether this represents a more generalized phenomenon associated with dysregulated gene expression. In this regard our ability to link promoter or gene body methylation (5mC or 5hmC) to the regulation of specific genes did not prove to be 100% consistent across genomic regions or cell lines.

We found that there were NO-regulated genes associated with increases or decreases in promoter 5mC and 5hmC in both cell types. For example, 5mC methylation increased at the promoter regions of GJC2 and AAK1 genes which were transcriptionally upregulated by NO. Interestingly, 5mC methylation was also increased at the promoter regions of the genes SYTL1 and PRR15L, which were transcriptionally downregulated by NO. At gene bodies, 5mC was largely associated with increased gene expression. The strongest association was the enrichment of 5mC at typical enhancers in MDA-MB-668 cells which correlated strongly to downregulation of associated genes. 5hmC is often found in active gene bodies, and linked to gene transcription or translation^64^. 5hmC is usually present at transcription start sites (TSS) of genes with high CpG promoters, marked by bivalent histone modifications, suggesting a role in regulating gene expression by modulating chromatin accessibility or inhibiting repressor binding^65^. We noted increases in gene expression correlated with 5hmC losses in typical enhancers (MDA-MB-468).

Although linking 5mC/5hmC to changes in gene expression in our models proved to be complicated, we did find that many of the genes transcriptionally regulated by NO in cell culture showed similar directional expression changes in hundreds of clinical samples of NOS2-expressing tumors. Moreover, the directional changes in these NO-regulated methylated genes correlated to decreased patient survival for patients harboring NOS2 expressing tumors. For decades it has been widely accepted that DNA methylation at CpG promoter regions is associated with transcriptional repression. Yet, numerous recent studies challenge this by demonstrating the persistent inability to attribute gene expression causality to methylation at specific DNA loci^66^. Several studies have shown an inverse correlation between DNA methylation of the first intron and gene expression across multiple tissues and species^67^. Numerous other studies have demonstrated that gain of promoter methylation is associated with increased gene expression^68–70^. For example, a large study of prostate cancers (1,117 samples) found that hypermethylated genes were strongly associated with increased gene expression^71^. Further complicating the issue are the multiple studies demonstrating that both transcription factor binding and changes in gene expression can in some cases precede DNA demethylation^72,73^. DNA methylation appears to be highly context specific, even in a single cancer type like TNBC, the focus of this study. The correlations between TET expression, DNA methylation, and tumor progression are highly dependent on both the TET isoform (1, 2, or 3) and the type of breast cancer. For example, TET1 expression and 5hmC levels are decreased in tumor samples from luminal A, luminal B, and HER2-positive subtypes compared to normal breast tissue samples and correlate with larger tumors, advanced stage, lymph node status, and poor patient survival^74–77^. Conversely, in TNBC, TET1 expression is increased in tumor tissue samples compared to normal breast tissue samples, and its high expression correlates with poor patient outcomes^78,79^. Taken together, we favor a more stochastic view of NO-mediated gene expression changes where NO inhibition of TET may predominantly lead to indiscriminate disruption of DNA methylation that cancers exploit to regulate tumor-permissive gene expression programs. This may ultimately benefit cancer cell survival by creating transcriptional diversity among populations of cells independent of their genetic transcriptomic background. This theory is somewhat supported by our data demonstrating that although NO-dependent transcriptomic changes were vastly different within two TNBC cell lines, similarities were noted at the level of tumor pathway analysis.

In conclusion, this study demonstrates that NO is an endogenous regulator of TET activity and DNA methylation. This represents a novel and unprecedented functional role for NO in regulating steady-state DNA methylation (and hydroxymethylation) levels. How changes in DNA 5mC/5hmC at specific loci regulate the expression of NO-responsive genes and how this mechanism synergizes with or antagonizes other canonical NO signaling mechanisms is still an open question. In cancer, further mechanistic studies are needed to fully understand the functional consequences of NO-mediated TET inhibition in relation to transcriptional malleability, transcriptional heterogeneity, and phenotypic plasticity; all associated with more aggressive tumors, worse patient prognosis, and resistance to chemotherapies. The findings presented herein have been in the context of cancers (breast), but we suspect that the fundamental discovery that NO inhibits TET enzymes to change DNA methylation patterns is a contributing factor to numerous diseases where there is dysregulated NO synthesis and aberrant DNA methylation patterns^80^. Moreover, this discovery raises the possibility that NO could regulate DNA methylation to control gene expression under physiological settings which should be explored further. Our previous work demonstrated that NO is an endogenous regulator of histone posttranslational modifications^26,27^ and mRNA methylation^28^, and here we show how NO regulates DNA methylation. Therefore, in addition to its canonical roles in cell signaling and gene expression, NO should be recognized as a dominant regulator of the epigenetic landscape^81^.

## Materials and Methods

### Chemicals

(Z)-1-[N-(2-aminoethyl)-N-(2-ammonioethyl)amino]diazen-1-ium-1,2-diolate (DETA/NO), (Z)-1-[N-[3-aminopropyl]-N-[4-(3-aminopropylammonio)butyl]-amino]diazen-1-ium-1,2-diolate (SPER/NO), 2-(N,N-Diethylamino)-diazenolate-2-oxide (Diethylamine nonoate) (DEA/NO) were each a gift from Dr. Joseph E. Saavedra (NCI). Sulfanilamide (SULF; prepared to 2% (w/v) in 5% HCl and filtered to remove trace particles), N-(1-naphthyl)ethylenediamine dihydrochloride (NEDD) (Riedel-de Haen, Germany) and prepared to 0.1% (w/v) in H_2_O and filtered to remove trace particles. Ammonium iron(II) sulfate hexahydrate ((NH_4_)_2_Fe(SO_4_)_2_•6H_2_O; purity 99.997%), α-ketoglutaric acid sodium salt (αKG (2-OG); purity > 98%), (+)-sodium L-ascorbate (ascorbic acid; purity > 98%), NaCl (purity ≥ 99%), aminoguanidine hydrochloride (purity ≥ 98%), 5-Azacytidine (AZA; purity ≥ 98%), 3-Hydroxypicolinic acid (3-HPA; purity ≥ 99%), ammonium citrate dibasic (purity ≥ 99%), bovine serum albumin (BSA), catalase (bovine liver) all obtained from Sigma-Aldrich. Dithiothreitol (DTT) (Bio-Rad). HEPES buffer (pH 7.2–7.5) (Gibco). Adenosine 5’-triphosphate disodium salt hydrate (Thermo Scientific). Cycloleucine (purity > 98%) (Chem Impex). N(G)-nitro-L-arginine methyl ester hydrochloride (L-NAME; purity > 99%) (Cayman Chemical). Amlodipine besylate (Major Pharmaceuticals). L-NG-Monomethylarginine, Acetate Salt (L-NMMA; purity ≥ 99%) (Santa Cruz Biotechnology).

### Cell culture and DETA/NO treatment

Both MDA-MB-231 and MDA-MB-468 cells were obtained from American Type Culture Collection (ATCC) and cultured in high glucose DMEM (Gibco #11995–065) supplemented with 10% fetal bovine serum (Gibco #26140–079) and 1% penicillin/streptomycin (Gibco #15140122) at 37°C in a humified atmosphere of 5% CO2. MDA-MB-231 cells expressing NOS2 were graciously provided by Dr. Sharon Glynn of the University of Galway, and were stably transduced with either the NOS2 lentiviral vector obtained from Origene, or an empty vector. The cells were cultured in RPMI 1640 with 5% L-glutamine (Millipore Sigma #R8758), 10% fetal bovine serum (FBS; Gibco), 1% penicillin/streptomycin (P/S; Gibco), selected with 2 μg/mL puromycin at 37°C with 5% CO_2_. To treat cells with the NO-donor DETA/NO, DETA/NO was first thawed on ice and diluted 1:1000 in 10 mM NaOH before measuring absorbance at λ = 250 nm to determine the concentration of the stock solution. The same procedure was used for NO donors SPER/NO and DEA/NO. For the low dose, chronic treatment, cells were plated in a 10 cm dish and treated with 50 μM or 100 μM DETA/NO for a total of 10 days, while passaging and re-treating every 2 days.

### Griess assay to detect NO via nitrite

The Griess assay for NO (nitrite) detection was conducted using media from NOS2-overexpressing cells. In each well of a 96-well plate, 100 μL of media was introduced, after which 50 μL of 2% (w/v) sulfanilamide in 5% HCl and 50 μL of 0.1% N-(1-Naphthyl)ethylenediamine dihydrochloride (NEDD) in H_2_O were added to the media. Plates were then incubated at 37°C for 40 minutes. The absorbance of the samples was measured at 540 nm and nitrite concentration was calculated using linear regression of sodium nitrite standards.

### Cell proliferation and invasion xCELLigence assays

Cell proliferation and invasion was measured using the Agilent xCELLigence Real-Time Cell Analysis system. For proliferation studies, 7,500 cells/well were plated onto e-plates and placed in the 37°C, 5% CO_2_ system to allow cell adherence. After 21 hours, if cells were treated, they received 100 μM DETA/NO and were placed back into the system for continued observation for 24 hours. For invasion studies, 50 μL Matrigel was first poured onto the e-plates. After allowing to harden, 20,000 cells/well were plated and allowed to adhere. After 25 hours, if cells were treated, they received 600 μM DETA/NO and were placed back into the system for continued observation for 24 hours.

### Cell-derived xenograft mouse studies

Mouse studies were performed by the Wink lab at the National Cancer Institute. Eight-week-old female athymic nude mice were supplied by the Frederick Cancer Research and Development Center Animal Production Area. After an initial period of 1–2 weeks, each mouse received an mammary fat pad injection of 750,000 MDA-MB-231 human triple-negative breast cancer (TNBC) cells overexpressing GFP. Following one week of tumor growth, mice were randomly assigned to control and treatment groups. The treatment group received the NOS inhibitor aminoguanidine at a concentration of 0.5 g/L in filter-sterilized drinking water. After 6 weeks, the mice were sacrificed and subjected to imaging. All animal protocols were approved and followed the principles outlined in the Guide for the Care and Use of Laboratory Animals by the Institute of Laboratory Animal Resources, National Research Council.

### Patient-derived xenograft mouse studies

PDX *in vivo* studies were conducted by the Chang Lab at Houston Methodist Hospital in 3 PDXs derived from primary human TNBC tumors: BCM-5998, BCM-3107, and BCM-3807. These PDXs were transplanted into the cleared mammary fat pad of mice. Upon reaching an average tumor volume of 150–250 mm^3^, the mice were randomly assigned to either the treatment or control group. For treatment, mice received intraperitoneal injections of L-NMMA (Santa Cruz Biotechnology) and amlodipine in sterile PBS (100 μL total/animal). L-NMMA was administered at 400 mg/kg on the first day and 200 mg/kg on subsequent days. Amlodipine (Major Pharmaceuticals NDC 0904-6371-61) was given at a dose of 10 mg/kg along with each L-NMMA treatment to counteract the elevated blood pressure associated with eNOS inhibition. The control group received sterile PBS via intraperitoneal injection (100 μL/animal). All animal procedures were approved by the Houston Methodist Hospital Research Institute Animal Care and Use Review Office.

### Western blot

Whole cell lysates were obtained using the RIPA Lysis Buffer System (Santa Cruz), which includes lysis buffer, phenylmethylsulfonyl fluoride, protease inhibitor cocktail, and sodium orthovanadate. The protein concentration was determined using the Lowry assay (Biorad DC protein assay). Subsequently, 30–40 μg of lysate was loaded into each well of a 10-well 10% Mini-PROTEAN TGX precast gel with Laemmli sample buffer and β-mercaptoethanol. Electrophoresis was conducted at 115 V for 1 hour. Protein transfer to a PVDF membrane was accomplished using the iBlot^™^ Transfer System (Invitrogen). The membrane was then blocked and incubated overnight at 4°C with the primary antibody in 5% milk in PBS-Tween. Following secondary antibody incubation, each blot was imaged using the FluorChem E system (ProteinSimple). Chemiluminescent substrate coating, either SuperSignal^™^ West Femto Maximum Sensitivity Substrate (Thermo Scientific) or SuperSignal^™^ West Pico PLUS Chemiluminescent Substrate (Thermo Scientific), was applied before imaging. Densitometry was conducted using Fiji (ImageJ). The list of antibodies used is provided below.

**Table:** 

Target protein	Concentration	Source/Isotype	Catalog Number
TET1	1:1000	Rabbit	GeneTex GTX124207
TET2	1.5 μg/mL	Mouse	Active Motif 61390
TET3	1:1000	Mouse	Abiocode M1092-3
ALKBH2	1:4000	Rabbit	Abcam ab154859
DNMT1	1:1000	Rabbit	CellSignaling 5032S
DNMT3a	1:1000	Rabbit	ABclonal A19659
DNMT3b	1:1000	Rabbit	ABclonal A11079
NOS2	1:1000	Rabbit	ABclonal A0312
β-Actin	1:1000	Rabbit	CellSignaling 4970S
Anti-Mouse Secondary	1:2000	Horse	CellSignaling 7076S
Anti-Rabbit Secondary	1:2000	Goat	CellSignaling 7074S

#### E. Coli expression/purification and i n vitro TET2 enzymatic assays

TET2 enzyme was expressed and purified from BL21 *E. Coli* (as described^33^) using a truncated 54.64 kD TET2 (1099–1936 with residues 1481–1843 replaced by a 15-residue GS linker). *In vitro* TET2 enzymatic assay was conducted by incubating 10 μM of an 8-nucleotide double-stranded DNA substrate (5’-CAC-XGGTG-3’, where X is 5mC, 5hmC, or 5fC) with 5 μM of purified TET2 and varying concentrations of Sper/NO or DEA/NO. The reaction was allowed to proceed for 1–3 hours at 37C in a 25 μL assay also containing 50 mM HEPES (pH 8.0), 100 mM NaCl, 100 μM Fe(NH4)2(SO4)2, 2 mM ascorbate, 1 mM DTT, 1 mM ATP, and 1 mM 2-KG. The DNA was desalted by adding 5 μL of AG^®^ 50W-X8 Cation Exchange Resin (BioRad, Cat # 143–5441) directly into the biochemical mixture and agitated followed by incubation for 5 min at room temperature. The samples were then centrifuged at 10,000 rpm for 2 min. 1 μL of sample was mixed with 1 μL of 3- Hydroxypicolinic Acid + Ammonium Citrate Dibasic matrix on a MALDI plate and the oxidized products were analyzed by MALDI-TOF mass spectrometry (Bruker-ultrafleXtreme^™^ MALDI-TOF/TOF spectrometer) using reflectron negative ionization mode and a mass range of 2400–2500.

### Dot blot for TET inhibition on genomic DNA

For *in vitro* time-dependent enzymatic activity assay on genomic DNA, 10 μM wild type TET2 (1099–1936 del-insert) containing 50 mM HEPES (pH 8.0), 100 mM NaCl, 100 μM Fe(NH_4_)_2_(SO_4_)_2_•6H_2_O, 2 mM ascorbate, 1 mM DTT, 1 mM ATP, and 1 mM 2-OG was incubated with 0–300 μM Sper/NO (freshly prepared) at room temperature for 25 min. The demethylase assay was initiated with 1 μg of genomic DNA, isolated from HEK293T cells, and incubated at 37°C for 3 hours. After incubation, ¼ volume of 2 M NaOH–50 mM EDTA was added before addition of 1:1 ice cold 2 M ammonium acetate. Immobilin-P PVDF membranes were cut to size, wet with MeOH for 20 sec, and equilibrated in TE buffer for 5 min, then assembled into a 96-well Bio-Dot microfiltration apparatus (Bio-Rad, Catalog #1706545). Each well was washed with 400 μL TE drawn through with gentle vacuum, and 400 ng of gDNA was loaded, followed by another TE wash. Membranes were blocked for 2 hours in 5% milk–TBST, washed 3× with TBST, and blotted at 4°C overnight with 1:3,000 anti-5hmC rabbit primary antibody (RRID-AB_10013602). Blots were then washed, incubated with secondary 1:5,000 goat anti-rabbit-HRP (Active Motif, cat. no. 15015) for 2 h. After another wash step, blots were imaged by chemiluminescence using VISIGLO HRP Chemiluminescent substrates A and B.

### ALKBH2 enzymatic assay

To prepare lysates, nuclear and cytosolic fractions were extracted and combined as described in (Fluorescence Probes of ALKBH2 Measure DNA Alkylation Repair and Drug Resistance Responses). 120 μL reactions were conducted in a 96-well plate at 37°C in 50 mM HEPES buffer (pH 8.0) containing 75 μM Fe(NH_4_)_2_(SO_4_)_2_•6H_2_O, 1 mM α-ketoglutarate, 2 mM Sodium Ascorbate, 50 μg/ml BSA, 0.4 mg/ml Catalase (Bovine liver, Sigma), 2 μM probe (m1a demethylase-detecting fluorescent probe 13p^35^), and 0–500 μM SperNO (for recombinant protein reaction) or 0–200 μM DETA/NO (for whole cell lysate reaction)). The time course of fluorescence activation was recorded after addition of 500 nM AlkBH2 (either recombinant or purified cell lysate). Probe was excited at 355 nm and emission collected at 460 nm for 1 hour for recombinant protein or 13 hours for cell lysate.

### MALDI-TOF TET2 assay

For in vitro enzymatic activity assays, wt-TET2 (1099–1936 del-insert) containing 50 mM HEPES (pH 8.0), 100 mM NaCl, 100 μM Fe(NH_4_)_2_(SO_4_)_2_•6H_2_O, 2 mM ascorbate, 1 mM DTT, 1 mM ATP, and 1 mM 2-KG was incubated with 150 μM Sper/NO (freshly prepared), at room temperature fro 25 min. The demethylase assay was initiated with 10 μM of double-stranded DNA substrate (5’-CAC-X-GGTG-3’, X-5mC, 5hmC, 5fC) and incubated at 37°C for 3 hr. Subsequently, the DNA was desalted by adding 8 μL of AG^®^ 50W-X8 Cation Exchange Resin (BioRad, Cat. #143–5441) directly into the biochemical mixture and agitated followed by incubation for 5 min at room temperature. The samples were centrifuged at 10,000 rpm for 2 min. The oxidized products were analyzed by MALDI-TOF mass spectrometry (AB SCIEX Voyager DE Pro and Bruker-ultrafleXtreme^™^ MALDI-TOF/TOF spectrometer) by spotting 1 μL of sample and then mixed with 1 μL of 3-Hydroxypicolinic Acid (3-HPA) matrix on MALDI plate.

To determine IC50 for Sper/NO against wt TET2, the assay mixture containing 5 μM protein was incubated with varying concentrations of Sper/NO (15 μM – 1,200 μM) in a buffer containing 50 mM HEPES (pH 8.0), 100 mM NaCl, 100 μM Fe(NH_4_)_2_(SO_4_)_2_•6H_2_O, 2 mM ascorbate, 1 mM DTT, 1 mM ATP and 100 μM 2OG for 20 min at room temp. Demethylation was initiated by adding 10 μM 8-nt double stranded 5mC DNA and further incubated at 37°C for 2 hr. The product DNA was processes as described above and by MALDI-TOF mass spectrometry. The values were fitted to the 4-S17 parameter non-linear regression algorithm (Y = Bottom + (Top - Bottom)/(1 + 10^((LogIC50 - X)*Hill slope))) of the GraphPad Prism software. X: log of dose or concentration; Y: response, decreasing as X increases; Top and bottom: upper and lower values of a given curve; logIC50: same log units as X; Hill Slope: Slope factor or Hill slope, unitless.

### 5mC and 5hmC ELISA

DNA was extracted from cells or tissues using the QIAGEN DNEasy Blood & Tissue kit and quantified using the BioTek Take3 system. Global %5mC and %5hmC were quantified by ELISA assay (Epigentek P-1030–96, P-1032–96). First, 100 ng DNA was bound to the bottom of each assay well. The wells were washed, and detection complex solution was added containing a 5mC or 5hmC antibody. After a 50-minute incubation, wells were washed, and color developer solution was added before measuring absorbance at 450 nm. %5mC and %5hmC were calculated as a proportion of the total DNA.

### Electron paramagnetic resonance (EPR)

TET2 reactions with NO were conducted in a hypoxic chamber with 5% CO_2_ and 95% N_2_. Solutions were degassed with N_2_ for one hour before bringing into the chamber. 28.8 μM purified truncated TET2 was incubated at 37°C with 500 μM DEA/NO, 50 μM double-stranded methylated DNA substrate (5’-CAC-5mCGGTG-3’), 100 μM ammonium iron(II) sulfate hexahydrate, 500 μM 2-oxoglutarate, 1 mM DTT, 2 mM ascorbate, 100 mM NaCl, 1 mM ATP, and 50 mM HEPES. Samples were flash frozen in liquid nitrogen either immediately after adding NO or 20 minutes after. X-band continuous wave EPR spectroscopy **(a)** at 77 K was conducted using a modified Varian E4 spectrometer equipped with a quartz finger dewar that operates at liquid nitrogen temperature, and **(b)** at 10 K using a Bruker ESP 300 spectrometer equipped with an Oxford Instruments ESR 910 continuous helium flow cryostat. Experimental parameters: for Fe(II)–NO signals 10 K, M.W. frequency 9.37 GHz, modulation amplitude 5 G; for DNIC spectra T = 77 K, M.W. frequency 9.135 GHz, modulation amplitude 10 G.

### RNA sequencing (RNA-seq) and analysis

Total RNA was isolated using the RNAqueous^™^-4PCR kit (Ambion) and treated with DNase I (Ambion) to avoid genomic DNA contaminations. Libraries were prepared with Kapa Hyper Stranded mRNA library kit (Roche). The libraries were pooled; quantitated by qPCR and sequenced on one SP lane for 101 cycles from one end of the fragments on a NovaSeq 6000. Fastq files were generated and demultiplexed with the bcl2fastq v2.20 Conversion Software (Illumina). Library preparation and sequencing was conducted by the University of Illinois at Urbana Champaign Roy J. Carver Biotechnology Center in triplicate, with a total of 476 million reads 100 nt in length. Read quality was assessed using MultiQC. Reads were aligned to hg38 GENCODE human genome (release 22) using STAR 2.5.2a^82^. Bam files were sorted using samtools^83^. Gene counts were generated using HTSeq^84^. Differential expression analysis was completed using edgeR v3.40.2^85^. Gene Set Enrichment Analysis was completed using OmicPath (https://github.com/CBIIT-CGBB/OmicPath).

### Oxidative reduced representation bisulfite sequencing (OxRRBS) and analysis

Genomic DNA was extracted and purified from cells using the DNEasy Blood & Tissue kit (QIAGEN) with RNase step. The libraries were prepared with the Ovation^®^ RRBS Methyl-Seq with TrueMethyl^®^ oxBS from Tecan. The libraries were pooled; quantitated by qPCR and sequenced on one S1 lane for 101 cycles from one end of the fragments on a NovaSeq 6000. Fastq files were generated and demultiplexed with the bcl2fastq v2.20 Conversion Software (Illumina). Oxidative reduced representation bisulfite sequencing library preparation and sequencing was performed by the UIUC core in duplicate with a total of 924 million reads 20–100 nt in length. Read quality was assessed using MultiQC^86^. Sequences were trimmed using TrimGalore (https://github.com/FelixKrueger/TrimGalore). Reads were aligned to hg38 using bowtie2 within Bismark (mapping efficiency ~ 62%) to count 5mC and 5hmC marks in a CpG context. Methylation analysis was performed using Bismark^87^ and Rnbeads^88^. Differential methylation analysis was performed using RnBeads, Dnmtools^89^, and MethylKit^90^. CpG annotation was performed with the annotatr package^91^ from BioConductor.

### Density functional theory simulations and modeling

Density functional theory simulations employed the Gaussian 16 code^92^ to investigate the stability of nitrosyl complexes of Fe(II) active site models of TET2. As per the study of Lu *et al*.,^41^ the wB97xD functional was employed as this Hamiltonian incorporates dispersion effects^93^, in conjunction with the def2-svp basis set^94^ for geometry optimizations and vibrational frequency calculation. For more accurate reaction energies, a single point calculation with a larger basis set – def2-tzvpp – was employed at the optimized stationary points. For the model complexes investigated all reasonable charge, spin and coordination states were examined. The results presented herein focus on the ground states identified for the proposed reaction intermediates. All complexes were fully optimized in the absence of any geometric or symmetry constraints. Quoted thermodynamic values assumed a temperature of 298.15 K and a pressure of 1 atm; enthalpic and entropic corrections used unscaled vibrational frequencies obtained at the wB97xD/def2-svp level of theory. An SMD continuum solvation model (solvent = water) was employed for closer congruence with experimental conditions^94^.

The DNIC was modeled into PDB file 4NM6^42^ using the modeling program COOT^95^. The model was energy minimized using REFMAC^96^ as implemented in CCP4i^97^. The NO bond length was restrained to 1.12 Å and the Fe-NO bond lengths restrained to 2.0Å.

## Data Availability

RNA-sequencing and RRBS-sequencing data are stored in the NCBI GEO repository under accession number GSE248151. Source data are provided with this paper.
